# *Nitrosophilus alvini* gen. nov., sp. nov., a hydrogen-oxidizing chemolithoautotroph isolated from a deep-sea hydrothermal vent in the East Pacific Rise, inferred by a genome-based taxonomy of the phylum “*Campylobacterota*”

**DOI:** 10.1371/journal.pone.0241366

**Published:** 2020-12-10

**Authors:** Taiki Shiotani, Sayaka Mino, Wakana Sato, Sayo Nishikawa, Masanori Yonezawa, Stefan M. Sievert, Tomoo Sawabe

**Affiliations:** 1 Laboratory of Microbiology, Faculty of Fisheries Sciences, Hokkaido University, Hakodate, Japan; 2 Department of Biology, Woods Hole Oceanographic Institution, Woods Hole, MA, United States of America; Columbia University, UNITED STATES

## Abstract

A novel bacterium, strain EPR55-1^T^, was isolated from a deep-sea hydrothermal vent on the East Pacific Rise. The cells were motile rods. Growth was observed at temperatures between 50 and 60°C (optimum, 60°C), at pH values between 5.4 and 8.6 (optimum, pH 6.6) and in the presence of 2.4–3.2% (w/v) NaCl (optimum, 2.4%). The isolate used molecular hydrogen as its sole electron donor, carbon dioxide as its sole carbon source, ammonium as its sole nitrogen source, and thiosulfate, sulfite (0.01 to 0.001%, w/v) or elemental sulfur as its sole sulfur source. Nitrate, nitrous oxide (33%, v/v), thiosulfate, molecular oxygen (0.1%, v/v) or elemental sulfur could serve as the sole electron acceptor to support growth. Phylogenetic analyses based on both 16S rRNA gene sequences and whole genome sequences indicated that strain EPR55-1^T^ belonged to the family *Nitratiruptoraceae* of the class “*Campylobacteria*”, but it had the distinct phylogenetic relationship with the genus *Nitratiruptor*. On the basis of the physiological and molecular characteristics of the isolate, the name *Nitrosophilus alvini* gen. nov. sp. nov. is proposed, with EPR55-1^T^ as the type strain (= JCM 32893^T^ = KCTC 15925^T^). In addition, it is shown that “*Nitratiruptor labii*” should be transferred to the genus *Nitrtosophilus*; the name *Nitrosophilus labii* comb. nov. (JCM 34002^T^ = DSM 111345^T^) is proposed for this organism. Furthermore, 16S rRNA gene-based and genome-based analyses showed that *Cetia pacifica* is phylogenetically associated with *Caminibacter* species. We therefore propose the reclassification of *Cetia pacifica* as *Caminibacter pacificus* comb. nov. (DSM 27783^T^ = JCM 19563^T^). Additionally, AAI thresholds for genus classification and the reclassification of subordinate taxa within “*Campylobacteria*” are also evaluated, based on the analyses using publicly available genomes of all the campylobacterial species.

## Introduction

The phylum “*Campylobacterota*” is a phylogenetically and ecophysiologically diverse bacterial group that consists of two classes, i.e., *Desulfurellia* (the former order *Desulfurellales*) and “*Campylobacteria*” (the former class *Epsilonproteobacteria*) [1]. While this phylum is widely recognized as a group including pathogenic microorganisms, e.g. *Helicobacter pylori* and *Campylobacter jejuni*, and many studies have focused on these pathogens [2, 3], an ever expanding number of non-pathogenic species have been identified which thrive as mesophiles or thermophiles in a wide range of natural environments (e.g., deep-sea hydrothermal fields, stratified ocean, terrestrial sulfidic caves, and oil fields) [4] where they are recognized as important players in biogeochemical cycles [1, 5]. Cultivation and characterization of these bacteria has also expanded our knowledge on the evolution and diversification of pathogenic relatives [6], biogeography [7], and the potential of biotechnological applications to mitigate global warming [8, 9].

At deep-sea hydrothermal vents, bacteria belonging to the phylum “*Campylobacterota*” are known as the dominant community members, including sulfide chimney structures where they can comprise up to 85% of the microbial biomass [10]. Taxonomically and metabolically diverse members of chemosynthetic “*Campylobacterota*” are responsible for the primary production [5, 11]. Ever since thermophilic “*Campylobacterota*” were first cultivated from hydrothermal vents [12], the number of culturable thermophilic members has increased with the refinement of cultivation conditions [13, 14]. Nevertheless, the described thermophilic species still account for only 14% of the total number of validly published species within “*Campylobacterota*”, and therefore there is still insufficient information on their genomes and intra-specific diversity. This also leaves the classification of thermophiles unresolved as almost all thermophilic families are composed of only a single genus, which were mostly retrieved from deep-sea hydrothermal vents.

The family *Nitratiruptoraceae* is one of the thermophilic groups within “*Campylobacteria*” that is frequently detected in deep-sea hydrothermal environments globally [5, 14]. This family consists so far of one validly described genus and species, *Nitratiruptor tergarcus*, isolated from the deep-sea hydrothermal chimney structure in the Mid-Okinawa Trough [15]. The recently described species “*Nitratiruptor labii*” was also isolated from the same deep-sea hydrothermal region [9]. In addition to the importance of *Nitratiruptoraceae* species in biogeochemical cycles [5], its potential for industrial applications has been described [16]. Isolation of the novel *Nitratiruptoraceae* species and elucidation of its physiological and genomic characteristics are both necessary to help understanding the diversity of this group and the evolutionary relationships within “*Campylobacteria”*.

16S rRNA gene sequences have been the universal molecular chronometer for microbial taxonomic affiliation for more than three decades, but this tool does not work well in classifying either closely related species [17, 18] or distantly related taxa [1]. The rapid advances in sequencing technology over the past decade have resulted in an increase in the amount of whole genome data and have brought significant opportunities to introduce robust and accurate criteria to improve microbial taxonomy [19]. One advance is the genome-based taxonomy based on the use of a large number of conserved core genes [20] and indices such as *in silico* DNA-DNA hybridization (DDH), average nucleotide identity (ANI), average amino acid identity (AAI), which refines phylogenetic analyses using genome sequence data. These classifiers enable the robust classification of novel species or genera, resulting in a more accurate microbial taxonomy [19]. Genome-based methods could also be effective in classifying members within the phylum “*Campylobacterota*”. However, the robust and accurate criteria using genome relatedness indices have not yet been fully evaluated for all species within “*Campylobacterota*”. In order to further expand the knowledge of their phylogenetic relationships and to propose a more robust classification methodology, establishment of clear classification criteria is needed to be evaluated.

Here, we report on the thermophilic campylobacterium, strain EPR55-1^T^, belonging to the novel genus *Nitrosophilus*, and evaluate the taxonomic assignment using a comprehensive approach based on whole genome sequence of the phylum “*Campylobacterota*”.

## Materials and methods

### Sample collection, enrichment and purification

The sample of a sulfide chimney structure was collected from the Bio9 deep-sea hydrothermal vent on the East Pacific Rise (9.83° N 104.28° W, water depth 2,511 m) by HOV *Alvin* during the AT26-23 scientific cruise aboard the R/V *Atlantis* in 2014. The interior part of the chimney sample was mixed anaerobically with 25 ml sterilized seawater containing 0.05% (w/v) neutralized sodium sulfide in 100 ml glass bottles (Schott Glaswerke) soon after HOV *Alvin* was recovered. The bottle was then tightly sealed with a butyl-rubber stopper under a gas phase of 100% N_2_ (0.2 MPa) and stored at 4°C until use. For enrichment, 100 μl of the resultant slurry was inoculated into 15 ml test tubes containing 3 ml MMJHS medium [21]. MMJHS medium contained 1 g NaHCO_3_, 1 g Na_2_S_2_O_3_·5H_2_O and 1 g NaNO_3_, 10 g S^0^ per liter MJ synthetic seawater. The medium was prepared under a H_2_/CO_2_ (80:20, v/v) gas phase (0.3 MPa). Growth of thermophiles was observed after one day at 55°C. Strain EPR55-1^T^ was isolated using the dilution-to-extinction technique [22] with MMJHS medium at 55°C. The purity was confirmed with a routine microscopic examination and by repeated partial sequencing of 16S rRNA gene using several PCR primers [23].

### Morphology and growth characteristics

Cells were observed using the ZEISS Axiophot microscope (Carl Zeiss Co., Oberkochen, Germany). For transmission electron microscopy, cells grown in MMJHS medium at 60°C in the late-exponential phase were stained with 1% (v/v) phosphotungstic acid. Micrographs were obtained using JEM-1011 transmission electron microscope (JEOL, Tokyo, Japan).

Growth was measured by direct cell counts after staining with 4′,6-diamidino-2-phenylindole [24]. The determination of the temperature range for growth was tested over the range 34-65°C (i.e. 34, 40, 50, 55, 57, 60 and 65°C) in 3 ml MMJHS medium. The pH range for growth was tested at 60°C in MMJHS medium buffered and adjusted to the required initial pH (i.e. pH 3.2, 5.4, 6.0, 6.6, 7.0, 7.7, 8.6 and 9.8). The range of NaCl concentrations for growth was tested over the range 0.8–4.0% (w/v) NaCl (i.e. 0.8, 1.6, 2.4, 3.2 and 4.0%, w/v) at 60°C in MMJHS medium.

The isolate was tested for its ability to grow on combinations of a single electron donor and acceptor. MJ synthetic seawater containing 0.1% (w/v) NaHCO_3_ was used as the basal medium. For testing the growth on hydrogen as an electron donor, H_2_/CO_2_ (80:20) was used as the gas phase. In an attempt to examine the growth on thiosulfate (0.1%, w/v), elemental sulfur (S^0^) (1%, w/v) or sodium sulfide (0.05% and 0.1%, w/v) as an electron donor, N_2_/CO_2_ (80:20) was used as the gas phase. Nitrate (0.1%, w/v), nitrous oxide (33%, v/v), thiosulfate (0.1%, w/v), sulfite (0.1, 0.01, 0.05 and 0.001%, w/v), elemental sulfur (1%, w/v), molecular oxygen (0.1 and 1%, v/v), nitrous oxide (33%, v/v) or fumarate (10 mM) were tested for potential electron acceptors. The presence or absence of growth was determined by microscopic observation.

Heterotrophic growth of strain EPR55-1^T^ was tested in MMJHS medium without NaHCO_3_ under a gas phase of 100% H_2_ (0.3 MPa), containing the following potential carbon sources: yeast extract, peptone, tryptone, casamino acids, D(+)-glucose, galactose, sucrose, fructose, lactose, maltose, starch (all 0.2%, w/v), formate, acetate, glycerol, citrate, tartrate, malate, succinate, propionate, lactate, oxalate, pyruvate (all 10 mM), methanol (0.05%, v/v), ethanol (0.1%, v/v) and 2-propanol (0.2%, v/v). In addition, to assess the utilization of these organic compounds as an energy source, substrates were added to MMJHS medium under a N_2_/CO_2_ (80:20) gas phase (0.3 MPa).

Potential nitrogen and sulfur sources required for growth were examined. To determine the nitrogen sources utilization, NH_4_Cl (0.025%, w/v), NaNO_3_ (0.1%, w/v) and NaNO_2_ (0.1%, w/v) were tested in MMJHS medium lacking all nitrogen sources, under a H_2_/CO_2_ (80:20) gas phase (0.3 MPa). In addition, utilization of N_2_ was examined under a H_2_/N_2_/CO_2_ (60:20:20) gas phase. In order to examine the sulfur sources for the growth of strain EPR55-1^T^, sulfate (0.42%, w/v), thiosulfate (0.1%, w/v), sulfite (0.1, 0.05, 0.01, 0.005 and 0.001%, w/v) and elemental sulfur (1%, w/v) were examined in MMJHS medium in which sulfur compounds were replaced with the chloride salts under an H_2_/CO_2_ (80:20) gas phase (0.3 MPa).

Susceptibility to antibiotics was tested in MMJHS medium containing ampicillin, chloramphenicol, kanamycin, streptomycin and rifampicin (all 100 μg ml^-1^).

### Molecular analysis based on 16S rRNA gene sequence

The 16S rRNA gene of strain EPR55-1^T^ was amplified by PCR using primers Eubac 27F and 1492R [23]. The nearly complete rRNA gene sequence (1,366 bp) was obtained by direct sequencing of both strands. The 16S rRNA gene sequence similarity analysis was conducted using BLAST search algorithm with all nucleotides [25]. To determine the phylogenetic position of the strain, the other “*Campylobacterota*” sequences were retrieved and aligned using Silva database [26] and Silva Incremental Aligner v1.2.11 [27], respectively. A phylogenetic tree was constructed using the neighbor-joining method [28] with the MEGA 7.0.21 software [29] using 1,166 bases. Bootstrap analysis was done using 1,000 replications to provide confidence estimates for the phylogenetic tree topologies.

### Genome sequencing and assembly

Genomic DNA of strain EPR55-1^T^ was extracted from the cells grown in MMJHS medium with Wizard genomic DNA purification kit (Promega, Madison, Wisconsin, USA) according to the protocol provided by the manufacturer. The genome was sequenced using Oxford Nanopore Technology (ONT) and Illumina sequencing platforms. The paired-end library for Illumina sequencing was generated using Nextera library preparation methods. Genome sequence was then performed on the MiSeq platform (2x300 bp paired-end). Read data from Illumina sequencing was trimmed with Platanus trim [30]. For the ONT sequencing, library was prepared using the Rapid Barcoding Sequence kit (Oxford Nanopore Technologies, Oxford, UK) according to the standard protocol provided by the manufacturer. The constructed library was loaded into the FlowCell (FLO-MIN106) on a MinION device and a 48-hour sequencing run with MinKNOW1.15.4 software was performed. After basecalling ONT reads with Guppy v1.1 (Oxford Nanopore Technologies) with following settings:—qscore_filtering and—calib_detect, basecalled reads were binned with Deepbinner [31]. Illumina reads were combined with ONT reads for coassembly with Unicycler version 0.4.7 [32], with default parameters. The genome was annotated using DFAST [33]. Metabolic pathways were analyzed by KEGG Automatic Annotation Server [34]. Orthologous genes between strain EPR55-1^T^ and *Nitratiruptor* members were determined with OrthoVenn [35] using protein sequences annotated by Prodigal [36].

### Calculation of genome sequence similarities

In order to determine the taxonomic positioning of the strain, genome-based taxonomic indexes were calculated. The *in silico* DDH values of strain EPR55-1^T^ against “*Nitratiruptor labii*” HRV44^T^ *Nitratiruptor tergarcus* MI55-1^T^ [14], *Nitratiruptor* sp. SB155-2 [6] and *Hydrogenimonas thermophila* EP1-55-1%^T^ [37] were calculated using the Genome-to-Genome Distance Calculator [38] with the BLAST^+^ alignment tool. Results were based on recommended formula 2, which is independent of genome length and is thus robust against the use of incomplete draft genomes.

To evaluate the genus-level AAI value of the phylum “*Campylobacterota*”, comprehensive AAI calculation was performed by using the aai.rb script (https://github.com/lmrodriguezr/enveomics). In addition, in order to further consider genus-level boundaries of the families *Nitratiruptoraceae* and *Nautiliaceae*, genome-wide ANI (gANI), alignment fractions (AF) [39], percentage of conserved proteins (POCP) [40], and the similarity of partial and/or complete 16S rRNA gene sequences were also calculated by ANIcalculator [41], the POCP.sh script developed by Harris et al. [42], and local blastn, respectively. A total of 160 “*Campylobacterota*” genome retrieved from NCBI RefSeq and GenBank prior to 15 April 2020 and “*Nitratiruptor labii*” genome were used for the analyses (S1 Table).

### Phylogenomic tree analyses based on whole genome and multilocus sequences of “*Campylobacterota*”

Phylogenomic tree was reconstructed using anvi’o v5.5 [43] based on protein sequences of 139 single-copy core genes (SCGs) from 160 genome sequences of members within “*Campylobacterota*”. The phylogenomics workflow (http://merenlab.org/2017/06/07/phylogenomics/) was followed to infer evolutionary associations between genomes. Briefly, the fasta files containing nucleotide sequences of genomes was used for generating the database of each genome (anvi-script-FASTA-to-contigs-db). We then identified an HMM profiles (anvi-get-sequences-for-hmm-hits) and extracted 139 SCGs proposed by Campbell et al. [44]. The amino acid sequences of 139 SCGs were then concatenated in a fasta file (anvi-get-sequences-for-hmm-hits). The optimal model for phylogenomic reconstruction was determined using Modelgenerator [45], and a ML tree was constructed using RAxML-NG version 0.9.0 [46] with LG+I+G4+F model. To further understand the reticulate evolution of “*Campylobacterota*”, a NJ tree was constructed using the same dataset using SplitsTree version 4.14.6 [47].

To deduce phylogenetic relationships among “*Campylobacterota*” species using a small set of genes, we conducted the multilocus sequence analysis (MLSA), which is a powerful method to elucidate genetic diversity [48] without whole genome sequencing. Nucleotide sequences of seven genes (*atpA*, *dnaK*, *glyA*, *gyrB*, *metG*, *pheS* and *tkt*) used in previous studies [7] were retrieved from 154 genome sequences of “*Campylobacterota*” including strain EPR55-1^T^ using the *in silico* molecular cloning software (In Silico Biology, Yokohama, Japan). Sequences of each genes were aligned using ClustalX version 2.1 [49] and then gaps were removed with a consideration of the reading frame. Gap-removed alignments of all genes were concatenated using Seaview [50]. NJ trees was constructed using SplitsTree based on concatenated amino acid sequences [47].

## Results

### Morphology and growth characteristics

**Fig 1:**
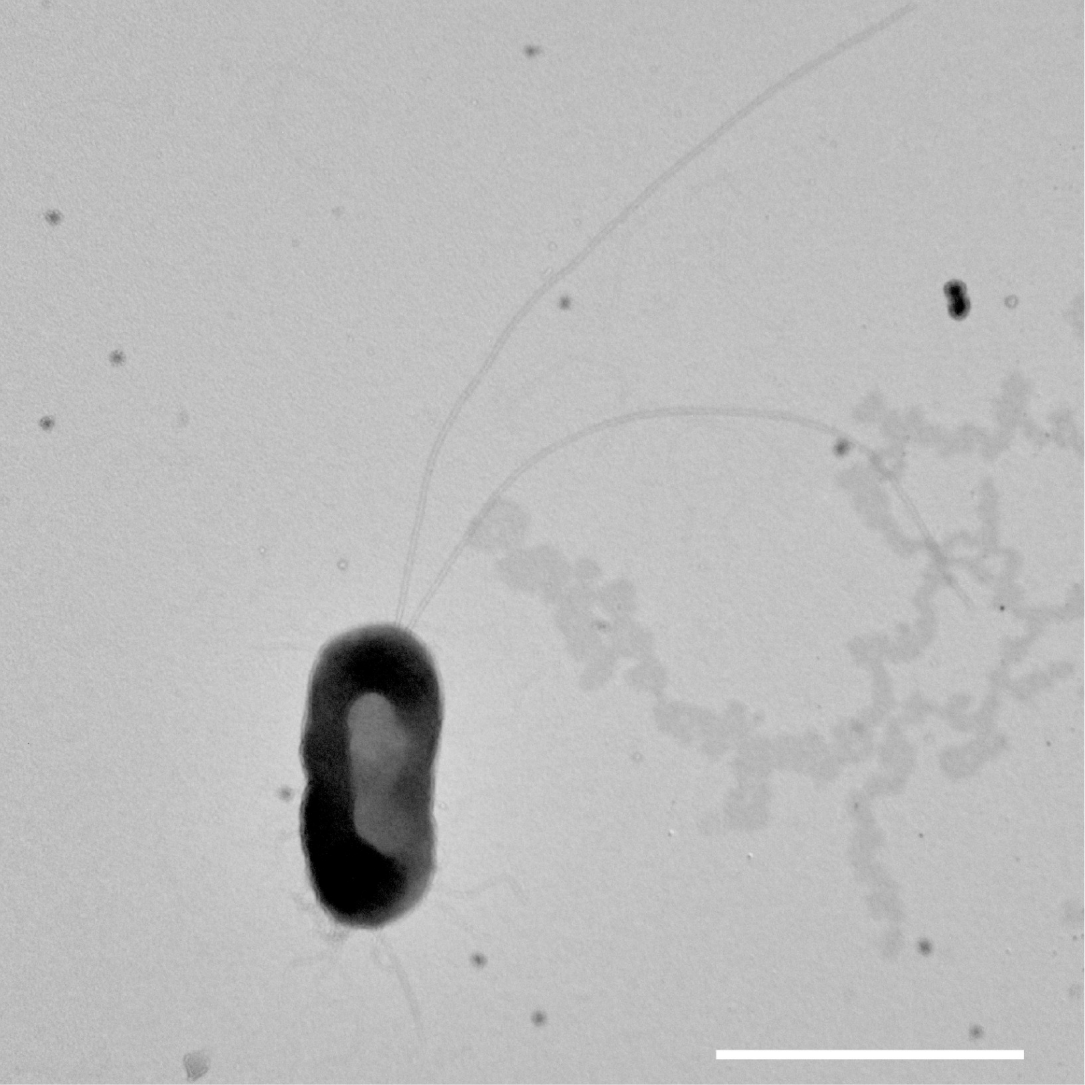
Electron micrograph of negatively stained cells of strain EPR55-1^T^. Scale bar represents 1.0 μm.

Cells of EPR55-1^T^ were Gram-negative rods (1.0 μm long and 0.5 μm in wide) (Fig 1). Cells were motile by means of flagella. Spore formation was not observed.

Strain EPR55-1^T^ grew at temperature between 50°C and 60°C, with optimum growth at 60°C. No growth was observed below 40°C or above 65°C. Growth occurred between pH 5.4 and 8.6, with optimum growth at pH 6.6. No growth was detected below pH 3.2 or above pH 9.8. Growth was observed NaCl concentrations between 2.4 and 3.2% (w/v), with optimum growth at 2.4%. No growth was observed at concentrations below 1.6% or above 4.0% (S1 Fig). Temperature, pH, and NaCl ranges for growth of strain EPR55-1^T^ were similar to those of “*Nitratiruptor labii”* HRV44^T^ [9] (Table 1).

**Table 1:** Comparison of physiological characteristics of EPR55-1^T^ with species of the families *Nitratiruptoraceae* and *Hydrogenionaceae*.

Characteristics	1	2	3	4	5
Origin	East Pacific Rise	Mid-Okinawa Trough	Mid-Okinawa Trough	Mid-Okinawa Trough	Central Indian Ridge
Temperature range (°C)	50–60	45–60	40–55	37–65	35–65
Optimum temperature (°C)	60	53	55	55	55
pH range	5.4–8.6	5.4–6.4	5.4–6.9	ND	4.9–7.2
Optimum pH	6.6	6.0	6.4	ND	5.9
NaCl range (%, w/v)	2.4–3.2	2.0–4.0	1.5–4.0	ND	1.6–5.6
Optimum NaCl (%, w/v)	2.4	2.5	2.5	ND	3.2
Electron donors	H_2_	H_2_,	H_2_	H_2_, S^2-^, S^0^, S_2_O_3_^2-^	H_2_
Electron acceptors	NO_3_^-^, N_2_O, S_2_O_3_^2-^, O_2_, S^0^	NO_3_^-^, N_2_O, S^0^, O_2_	NO_3_^-^, O_2_, S^0^^†^	NO_3_^-^, O_2_	NO_3_^-^, O_2_, S^0^
Carbon sources other than CO_2_	-	-	-	ND	-
Nitrogen sources	NH_4_^+^	NO_3_^-^, NH_4_^+^	NO_3_^-^, NH_4_^+^	ND	NO_3_^-^, NH_4_^+^
DNA G + C content	37.7	33.4	36.9	39.7	33.5

-, negative; ND, not determined.

†S^0^ could not serve as a sole electron acceptor to support growth.

1, Strain EPR55-1^T^; 2, “*Nitratiruptor labii*” HRV44^T^ [9]; 3, *Nitratiruptor tergarcus* MI55-1^T^ [14]; 4, *Nitratiruptor* sp. SB155-2 [6]; 5, *Hydrogenimonas thermophila* EP1-55-1%^T^ [37].

Strain EPR55-1^T^ was only able to use H_2_ as electron donor. Nitrate (0.1%, w/v), N_2_O (33%, v/v), thiosulfate (0.1%, w/v), elemental sulfur (1%, w/v) and molecular oxygen (0.1%, v/v) were able to serve as the sole electron acceptors. The isolate could not utilize any organic compounds as energy or carbon sources. These results indicated that strain EPR55-1^T^ was a strictly hydrogen-oxidizing thermophilic chemolithoautotroph. The isolate was able to use ammonium as its sole nitrogen source and utilization of N_2_ was not observed. Strain EPR55-1^T^ utilized thiosulfate, sulfite (0.01 to 0.001%, w/v) and elemental sulfur as sulfur sources. None of the chemosynthetic “*Campylobacterota*” isolated so far are reported as possessing the ability to utilize sulfite as its sulfur source. Strain EPR55-1^T^ was sensitive to ampicillin, chloramphenicol, kanamycin, streptomycin and rifampicin.

### Phylogenetic analysis based on 16S rRNA gene sequences

**Fig 2:**
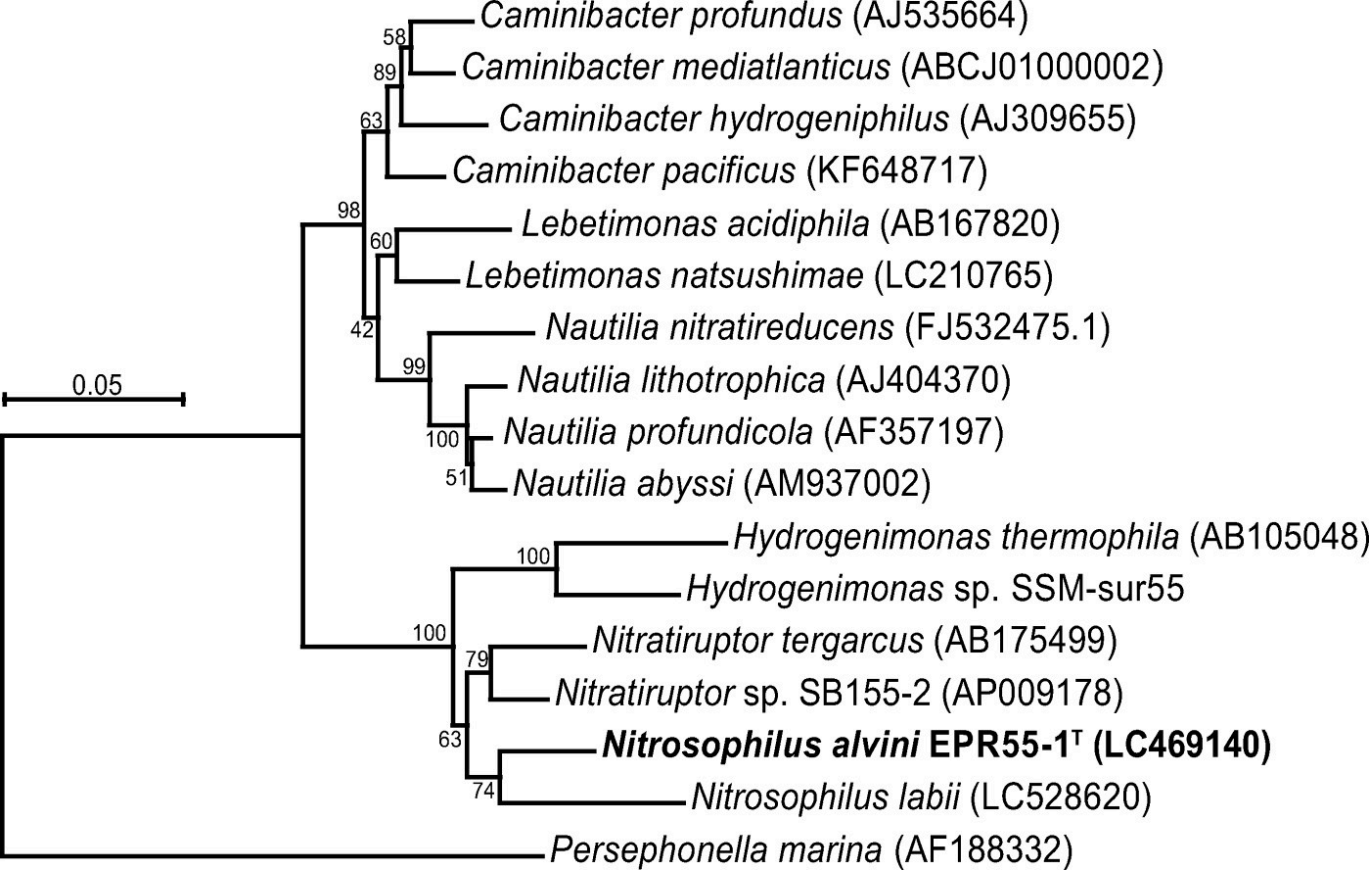
Phylogenic tree based on 16S rRNA gene sequences. Phylogenetic tree of the members of thermophilic “*Campylobacterota*”, inferred by the neighbor-joining algorithm using 1,166 homologous sequence positions. Numbers at branches are bootstrap values (%) based on 1,000 replicates.

With a nearly full length of 16S rRNA gene sequence of strain EPR55-1^T^ as a query in BLAST search, 96.0%, 94.3%, 93.0%, and 93.0% similarity were estimated with “*Nitratiruptor labii*” HRV44^T^, *Nitratiruptor* sp. SB155-2, *Nitratiruptor tergarcus* MI55-1^T^, and *Hydrogenimonas thermophila* EP1-55-1^T^, respectively, indicating that strain EPR55-1^T^ may be a new species of *Nitratiruptor* or even a member of newly described genus with strain HRV44^T^. The phylogenetic analysis showed that strain EPR55-1^T^ was closely related to the *Nitratiruptor* species (Fig 2).

### Genome properties

**Fig 3:**
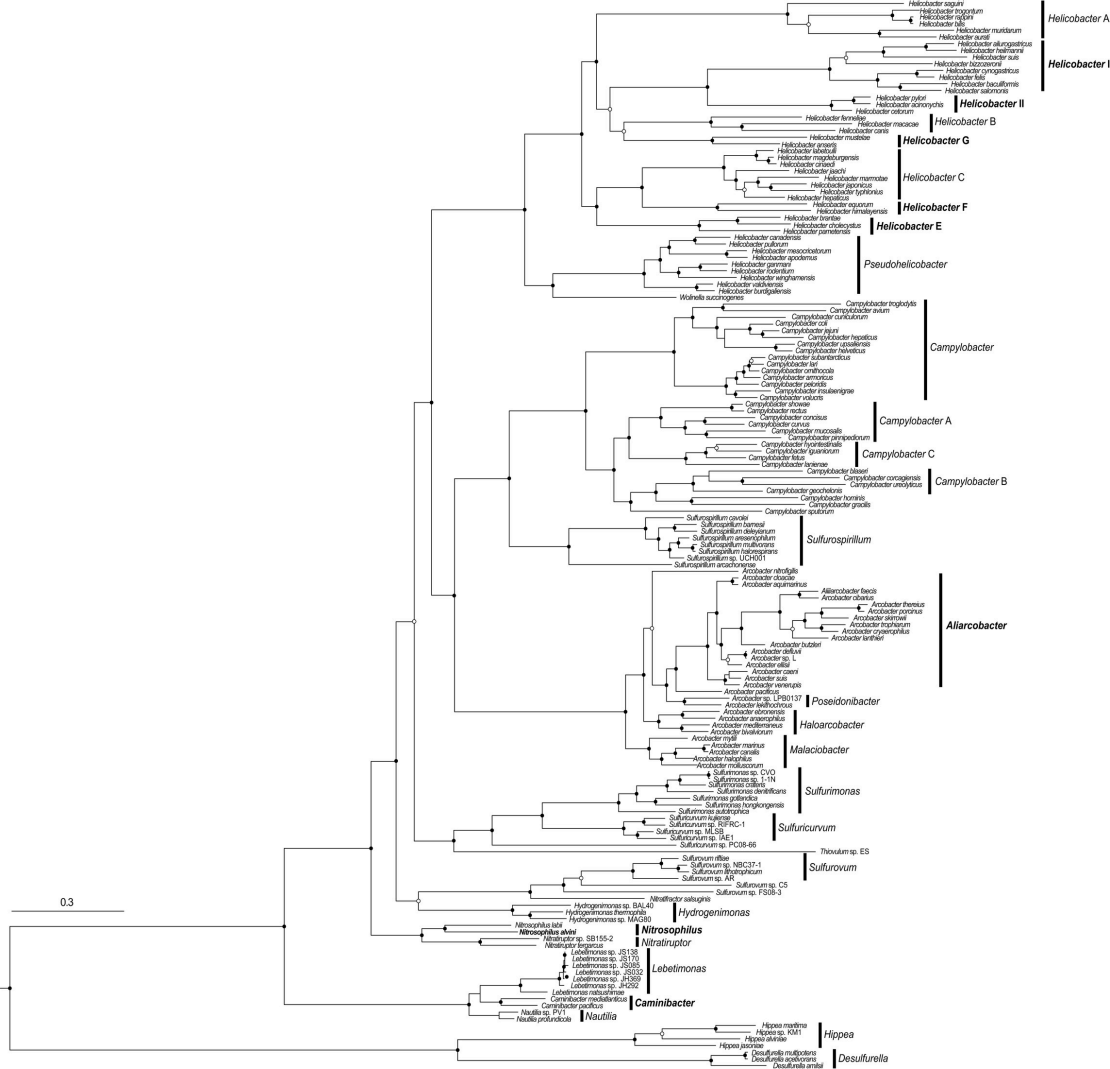
Maximum likelihood tree of 160 members within “*Campylobacterota*”. Maximum likelihood tree was constructed based on amino acid sequences of 139 SCGs using RAxML. Support of internal nodes was calculated using 100 bootstrap iterations. Bootstrap support of 100% and >75% are denoted by solid and hollow, respectively.

Hybrid genome assembly with Unicycler resulted in a single complete circular contig with a length of 1,807,889 bp. Of the 1,833 genes predicted, 1,783 were coding sequences (CDSs), 41 tRNA genes, and 3 set of rRNA genes (Fig 3). These values were comparable to those of closely rerated *Nitratiruptor* isolates; “*Nitratiruptor labii*” HRV44^T^ (1,990,315 bp and 2,050 CDS without a plasmid) *Nitratiruptor tergarcus* MI55-1^T^ (1,894,691 bp and 1,935 CDSs) and *Nitratiruptor* sp. SB155-2 (1,877,931 bp and 1,857 CDSs) (Nakagawa et al., 2007). The G + C content was 37.7%, which is similar to that of *Nitratiruptor* sp. SB155-2 (39.7%) (Table 1).

### Taxonomic placement of EPR55-1^T^ on the basis of genomic analyses

The *in silico* DDH values of strain EPR55-1^T^ against “*Nitratiruptor labii*” HRV44^T^, *Nitratiruptor tergarcus* MI55-1^T^ and *Hydrogenimonas thermophila* EP1-55-1%^T^ were 18.7%, 18.1% and 17.4%, respectively, well below a threshold of 70% *in silico* DDH used for the definition of bacterial species [38]. In addition, ANI values of strain EPR55-1^T^ against “*Nitratiruptor labii*” HRV44^T^, *Nitratiruptor tergarcus* MI55-1^T^ and *Hydrogenimonas thermophila* EP1-55-1%^T^ were 77.5%, 71.4% and 70.4%, respectively, well below the species threshold (95.0%) [51]. These results support the proposal that the isolate is a novel species within the class “*Campylobacteria*”. AAI values of the novel isolate against “*Nitratiruptor labii*” HRV44^T^, *Nitratiruptor tergarcus* MI55-1^T^, *Nitratiruptor* sp. SB155-2 and *Hydrogenimonas thermophila* EP1-55-1%^T^ were 69.9%, 64.1%, 63.7% and 59.4%, respectively, which fall within the threshold for genus-level differentiation (60–80%) [52]. The gANI and AF of strain EPR55-1^T^ against the closely related species were 73.85% and 0.53 to “*Nitratiruptor labii*” HRV44^T^, 71.45% and 0.38 to *Nitratiruptor tergarcus* MI55-1^T^, 71.32% and 0.38 to *Nitratiruptor* sp. SB155-2, respectively (S2 and S3 Tables). These values below the genus level threshold (gANI value of 73.98 (mean) and 73,11 (median) [39], AF value of 0.33 (mean) and median (0.345) [39]) are indicative of genus-level differentiation of strain EPR55-1^T^ with strain HRV44^T^, though POCP values of strain EPR55-1^T^ against the all three relative strains were >70.1%, higher than genus threshold (≥50%) (S4 Table).

The Venn-diagram showed the presence of a conserved core set of 1,270 gene clusters that are shared by all *Nitratiruptor* genomes, representing more than a half of the proteins in each strain. In addition, strain EPR55-1^T^, HRV44^T^, MI55-1^T^ and SB155-2 possessed 215, 345, 250 and 245 singletons, respectively (S2 Fig).

### Comparison of phylogenomic and genomic distance within “*Campylobacterota*”

AAI analysis between 160 genomes, which vary extensively within “*Campylobacterota*”, illustrates that there are genera, which need to be considered reclassification. AAI, gANI, AF, and POCP values between type strains of the family *Nautiliaceae*, *Nautilia profundicola* AmH^T^, *Caminibacter mediatlanticus* TB-2^T^, *Cetia pacifica* TB-6^T^, and *Lebetimonas natsushimae* HS1857^T^, were 71.6–74.2%, 76.1–78.0%, 0.52–0.81, and 76.02–84.0%, respectively, within or higher than the genus demarcation given by previous studies (S2–S4 Tables). The phylogenomic analyses based on both SCGs and MLSA genes also showed these strains could be regarded as one clade (Figs 3 and S4 and S5). Although the genome-based taxonomy indicated these strains could be considered to the species belonging the same genus, the current inter-genus 16S rRNA gene sequence identities of these strains were below 94.5% [53] with the exception of *Cetia pacifica* TB-6^T^ which showed >95% identity to all type strains of the genus *Caminibacter* (S5 Table).

**Fig 4:**
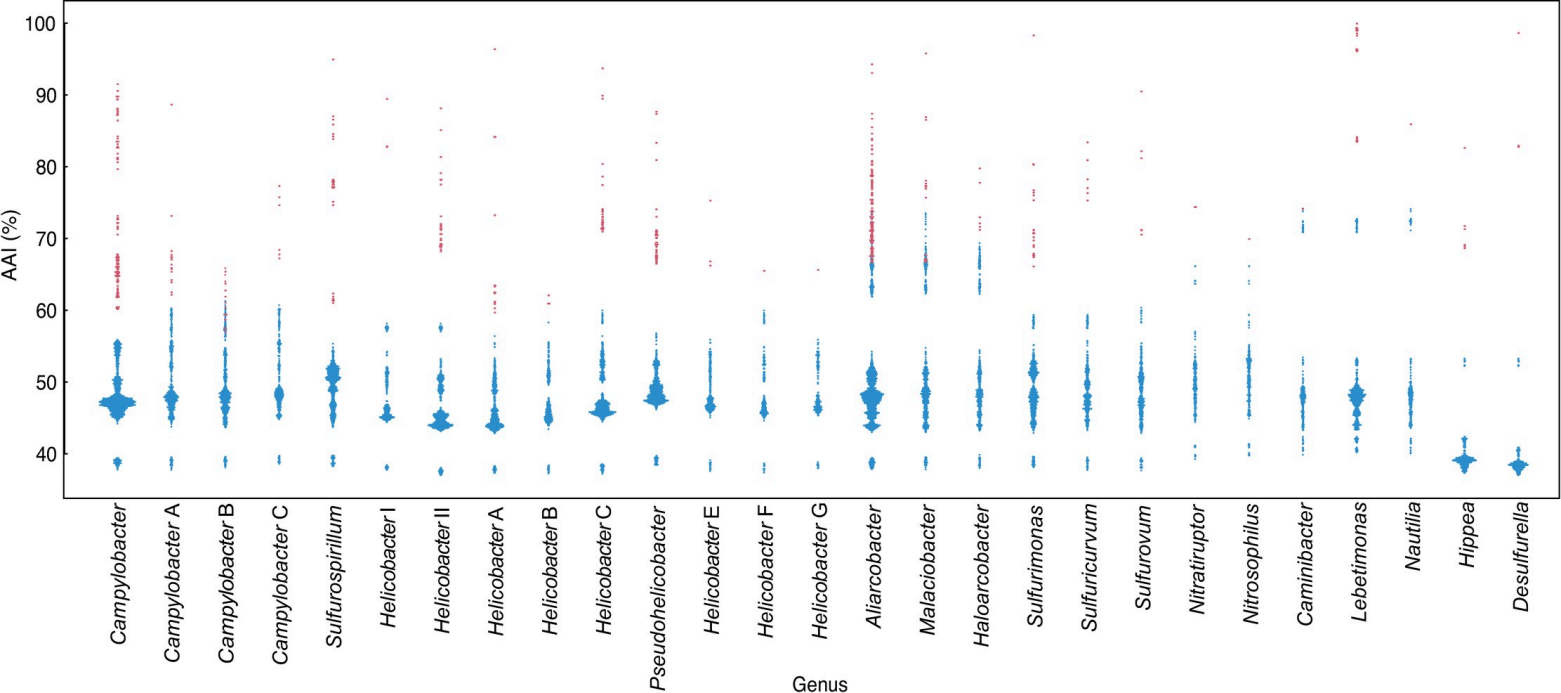
The beeswarm based on the AAI values. Beeswarm plots showing the AAI values between species of “*Campylobacterota*”. Each dot shows a comparison between species. Comparisons between species within reclassified same genus and between species of different genera are colored in red and blue, respectively. Genera consisting of more than two species were shown in this figure.

In addition to the thermophilic taxa, some differences compared to the current classification were observed for the genus *Helicobacter* and the family *Arcobacteraceae* [54]. *Helicobacter pametensis* and *Helicobacter cholecystus*, *Helicobacter brantae* showed low AAI values against other *Helicobacter* species (48.5–55.8%). Similarly, *Helicobacter equorum* and *Helicobacter himalayensis*, and *Helicobacter anseris* and *Helicobacter mustelae* showed AAI values below the genus threshold [52] against other *Helicobacter* species (50.0–59.9% and 49.8–55.9%, respectively). Phylogenomic analyses also showed that these six species formed new three clades (*Helicobacter* E F, and G). Additionally, *Helicobacter pylori*, *Helicobacter acinonychis* and *Helicobacter cetorum*, currently belonging to the *Helicobacter* clade [1], showed lower AAI values than the genus threshold against other current *Helicobacter* clade species (57.0–58.1%). In the both NJ and ML phylogenomic trees on the basis on 139 SCGs, the current *Helicobacter* clade branched to two clades (*Helicobacter* I and II) (Figs 3 and S4). Same branching patterns were also observed in the NJ tree based on amino acid sequences of MLSA genes (S5 Fig). In the genera of the family *Arcobacteraceae*, AAI values between species belonging to different genera *Aliarcobacter*, *Poseidonibacter*, *Malaciobacter*, *Arcobacter*, *Halarcobacter* were 61.9–78.7%, indicating they could be regarded as different species of the same genus. The large clade consisting of these genera was also identified by phylogenomic analyses based on both SCGs and MLSA genes (Figs 4 and S4 and S5). However, some species showed the inter-genus 16S rRNA gene sequence identities below 94.5% [53] (S5 Table), indicating differentiation at genus level. AAI values among *Campylobacter* B were relatively lower (57.02–65.86%), possibly due to the low degree of relatedness between *Campylobacter* B species (<94.5% 16S rRNA gene similarities).

Based on results of phylogenomic analyses, the AAI values between species belonging to same clades or the different clades were evaluated (Fig 4). The minimum AAI value between species belonging to same clades was 59.7% (*Helicobacter muridarum* ST1^T^ vs *Helicobacter saguini* MIT 97-6194^T^), and the maximum AAI value between species belonging to different clades was 61.9% (*Campylobacter geochelonis* RC20^T^ vs *Campylobacter hominis* ATCC BAA-381^T^) with the exception of *Nitratiruptoraceae*, *Nautiliaceae*, and *Arcobacteraceae* whose 16S rRNA gene sequence similarities were not reflected in the genome relatedness. These values indicated the AAI threshold for genus demarcation of “*Campylobacterota*” was about 60–62%, though there are some exceptions.

## Discussion

### Comparison of strain EPR55-1^T^ with related species

Strain EPR55-1^T^ was the first *Nitratiruptoraceae* species isolated from the East Pacific Rise. The strain shows some physiological differences from other *Nitratiruptoraceae* isolates, and represents the only *Nitratiruptoraceae* species which is able to utilize thiosulfate and sulfite as its sole electron acceptor and sulfur source, respectively. The ability to utilize sulfite has also never been reported in any other thermophilic campylobacterial species. The strain EPR55-1^T^ possessed lophotrichous flagella, unlike the monotrichous and amphitrichous flagella of “*Nitratiruptor labii*” and *Nitratiruptor tergarcus*, respectively [9, 14]. 16S rRNA gene sequence similarities, gANI, and AF values of the strain against closely related species suggested that strain EPR55-1^T^ designate the strain as a novel genus with strain HRV44^T^. The strain EPR55-1^T^ therefore represents a novel genus within a new genus of the family *Nitratiruptoraceae*, for which the name *Nitrosophilus alvini* gen. nov., sp. nov. is proposed.

### Proposed reclassifications among “*Campylobacterota*”

The 16S rRNA gene sequence similarity between of strain EPR55-1^T^ and HRV44^T^ was above 95%. Additionally, phylogenetic trees based on sequences of 16S rRNA gene, 139 SCGs, and MLSA genes showed that strain EPR55-1^T^ and HRV44^T^ formed a distinct branch from the genus *Nitratiruptor*. Considering the relationship between strain EPR55-1^T^ and “*Nitratiruptor labii*” HRV44^T^, we propose that “*Nitratiruptor labii*” should be transferred to the genus *Nitrosophilus* as a new combination, *Nitrosophilus labii* comb. nov.

While thermophilic species within the genera *Nautilia*, *Caminibacter*, *Cetia*, and *Lebetimonas* exhibited genome indexes where values exceeded the genus thresholds proposed in previous studies, their pairwise similarities of 16S rRNA gene sequences were below 94.5% except for similarities obtained by the comparison between *Cetia pacifica* TB-6^T^ and *Caminibacter* species. 16S rRNA gene-based and genome-based phylogenetic trees also indicated *Cetia pacifica* formed a clade with members of the genus *Caminibacter*. Consequently, we propose that *Cetia pacifica* should be transferred to the genus *Caminibacter* as a new combination, *Caminibacter pacificus* comb. nov.

In the genus *Helicobacter*, results of both AAI comparison and phylogenomic analyses indicate that *Helicobacter pametensis*, *Helicobacter cholecystus*, *Helicobacter equorum*, *Helicobacter himalayensis*, *Helicobacter anseris*, and *Helicobacter mustelae* could be regarded as three novel genera of *Helicobacteraceae* (*Helicobacter* E, F, and G). In addition, our results also suggest that *Helicobacter pylori*, *Helicobacter acinonychis* and *Helicobacter cetorum* might be differentiated from current genus *Helicobacter* and be representatives of a novel genus (*Helicobacter* II). These results are sufficient to propose the reclassification of the genus *Helicobacter* into eight genera (*Helicobacter* I, II, A, B, C, E, F, and G) updating the previously suggested grouping [1, 55]. When considering their habitat, *Helicobacter* I and II, and A to G including “*Pseudohelicobacter*” [55] are characterized as gastric and enteric genera, respectively [56]. In addition, based on the reclassification of the genus *Helicobacter*, physiological characteristics of 44 species were compared at the clade level (S6 Table). Since diverse physiological characteristics were observed in each clade, it is difficult to classify *Helicobacter* species by conventional method based on both physiological characteristics and 16S rRNA gene sequences. The classification method based on the genome sequences is therefore necessary to robustly classify the genus *Helicobacter*.

The historical genus *Arcobacter* was recently reclassified into six genera, *Arcobacter*, *Aliarcobacter*, *Pseudoarcobacter*, *Malaciobacter*, *Halarcobacter*, and *Poseidonibacter*, based on comprehensive phylogenetic approaches using genomic relatedness indices, housekeeping genes, core genomes, and the 16S rRNA gene [54]. This reclassification seems to be orversubdivided by comparing other genera within “*Campylobacterota*”, as AAI values between species belonging to different genera within family *Arcobacteraceae* were much higher level than those of genera within other families and as also refuted by On et al [57] (Fig 4). However, when considering the similarities of 16S rRNA gene sequences among these species, it is reasonable to maintain the multiple genera in *Arcobacteraceae* (S5 Table). Members of the genus *Pseudoarcobacter* formed a clade with members of the genus *Aliarcobacter*, which may be incorporated into the *Aliarcobacter* (Figs 3 and S4 and S5).

### Availability of MLSA for phylogenomic assignments of “*Campylobacterota*”

In the MLSA, selection of protein-coding genes and their number often vary between each taxon, and therefore common recommendations are still not in place [58]. For example, in the genus *Vibrio*, nine genes are used for describing the vibrio clades [59], and the number of MLSA genes for identifying the species could be reduced to four [60]. In addition, genes used for MLSA of pathogenic “*Campylobacteria*” vary between species (https://pubmlst.org/databases/). It is therefore necessary to establish a universal MLSA scheme for all “*Campylobacterota*” species in order to accurately reflect taxonomic relationships. The NJ trees on the basis of amino acid sequences of seven MLSA genes identified in the present study showed a similar topology to those on the basis of the whole genome sequences, suggesting that MLSA could reconstruct the taxonomic relationships within “*Campylobacterota*” as accurately as the whole genome analysis. However, the topology of NJ tree and decomposition network based on nucleotide sequences of MLSA genes differed from the taxonomic results based on amino acid sequence. Considering our results the MLSA based on amino acid sequences appears to be an effective tool to classify the novel species within the phylum “*Campylobacterota*” when whole genome sequence information in not available.

### Genome-based taxonomic scheme for the phylum “*Campylobacterota*”

16S rRNA gene sequencing is widely recognized and useful at the first step in the identification of the isolates. However, when the similarity between novel strains and its closest relatives are at around 94–96%, additional genome-based analyses are required to decide the if strain represents a novel species or a novel genus. Although the families *Nautiliaceae*, *Nitratiruptoraceae*, and *Arcobacteraceae* have relatively higher inter-genus AAI genome similarities, extensive comparison of AAI values within “*Campylobacterota*” showed that the genus threshold of “*Campylobacterota*” could be 60–62%, correlating well with the branching pattern of phylogenetic trees based on whole genomes. This threshold value corresponds to the observed genus-level differentiation (60–80% AAI) [52]. However, due to the genomic diversity of “*Campylobacterota*”, the AAI-based approach alone may be insufficient to classify a novel isolate at the genus level. In addition to the 16S rRNA-gene based phylogeny, constructing the phylogenetic trees based on SCGs and/or MLSA gene sequences is helpful to determine the taxonomic position of new isolates. When the strain forms clade with close relatives, the strain is classified in the same genus as its relatives. When a novel strain forms a clade with undescribed relatives, the strain could be classified as a novel genus.

### Description of *Nitrosophilus* gen. nov.

*Nitrosophilus* (Ni.tro.so.phi'lus. L. masc. adj. *nitrosus*, full of natron, here intended to mean nitrate and nitrous oxide; N.L. masc. adj. *philos*, loving, friendly to; N.L. masc. n. *Nitrosophilus* nitrate- and nitrous-oxide-loving, referring to the use of nitrate and nitrous oxide as a sole electron acceptor.

Cells are rod-shaped, motile and stain Gram-negative. Anaerobic to microaerobic. Strictly chemolithoautotrophic. Thermophilic, adapted to the salinity of the ocean. On the basis of 16S rRNA gene and single-copy core-gene analyses, the genus *Nitrosophilus* belongs to the family *Nitratiruptoraceae* within the class “*Campylobacteria*”. The type species is *Nitrosophilus alvini*.

### Description of *Nitrosophilus alvini* sp. nov.

*Nitrosophilus alvini* (al.vi’ni. N.L. gen. masc. n. *alvini* from the name of the HOV *Alvin* which collected the deep-sea hydrothermal samples harbouring this strain).

Cells are Gram-negative, motile, and rod-shape. The temperature range for growth is at 50–60°C (optimum 60°C). The pH range for growth is pH 5.4–8.6 (optimum 6.6). NaCl concentration range for growth is 2.4–3.2% (w/v) (optimum 2.4%). Strain EPR55-1^T^ is hydrogen-oxidizing, facultatively anaerobic and chemolithoautotrophic with molecular hydrogen as its sole electron donor and with nitrate, nitrous oxide, thiosulfate, molecular oxygen or elemental sulfur as its sole electron acceptors. Ammonium is utilized as its sole nitrogen source. Thiosulfate, sulfite or elemental sulfur are utilized as its sole sulfur source. The complete genome size is 1,807,889 bp. The G + C content of DNA is 37.7%. The type strain, EPR55-1^T^ (= JCM 32893^T^ = KCTC 15925^T^), was isolated from a deep-sea hydrothermal vent in the East Pacific Rise.

### Description of *Nitrosophilus labii* comb. nov.

Basonym: *Nitratiruptor labii* Fukushi et al., 2020 [9].

The description is the same given by Fukushi et al. (2020) [9]. The type strain is HRV44^T^ (= JCM 34002^T^ = DSM 111345^T^).

### Description of *Caminibacter pacificus* comb. nov.

Basonym: *Cetia pacifica* Grosche et al., 2015 [12].

The description is the same given by Grosche et al. (2015) [12]. The type strain is TB-6^T^ (= DSM 27783^T^ = JCM 19563^T^).

## Supporting information

S1 Fig Growth rates of strain EPR55-1^T^. Growth rates of temperature (a), pH (b) and NaCl concentration (c) in MMJHS medium.
(TIF)

S2 Fig Graphical circular map of the strain EPR55-1^T^ genome. Tracks from inside to outside are as follows: GC skew, G + C content, rRNA, reverse strand CDS, and forward strand CDS (color by COG categories).
(TIF)

S3 Fig Venn diagram of orthologous gene clusters among the genera *Nitratiruptor* and *Nitrosophilus*. This Venn diagram represents shared or unique orthologous gene clusters between EPR55-1^T^, HRV44^T^, *Nitratiruptor tergarcus* MI55-1^T^ and *Nitratiruptor* sp. SB155-2.
(TIF)

S4 Fig Consensus NJ tree of “*Campylobacterota*” based on SCGs. The phylogenomic tree was constructed based on 139 SCG protein sequences retrieved from 160 genomes belonging to “*Campylobacterota*”. The emended and newly proposed taxa in the phylum “*Campylobacterota*” (*Nitrosophilus*, *Caminibacter*, *Helicobacter* I, II, E, F, G, and *Arcobacter*) were shown in bold.
(TIF)

S5 Fig Consensus NJ tree of “*Campylobacterota*” based on MLSA genes. The phylogenetic tree was constructed based on amino acid sequences of MLSA genes (i.e. *atpA*, *dnaK*, *glyA*, *gyrB*, *metG*, *pheS* and *tkt*) retrieved from 154 members of “*Campylobacterota*”. *Arcobacter cloacae* F26, *Arcobacter ebronensis* CECT 8441, *Arcobacter mediterraneus* F156-34, *Campylobacter mucosalis* DSM 21682, *Helicobacter bizzozeronii* CIII-1, *Hydrogenmonas* sp. MAG80, and *Thiovulum* sp. ES were excluded because of lack of at least one MLSA gene sequence.
(TIF)

S1 Table Genome data used in this study. (XLSX)

S2 Table gANI values between species belonging to the families *Nitratiruptoraceae* and *Nautiliaceae*. (XLSX)

S3 Table AF values between species belonging to the families *Nitratiruptoraceae* and *Nautiliaceae*. (XLSX)

S4 Table POCP values between species belonging to the families *Nitratiruptoraceae* and *Nautiliaceae*. (XLSX)

S5 Table Similarities of 16S rRNA gene sequences between species belonging to the families *Campylobacteraceae*, *Sulfurospirillacee*, *Helicobacteraceae*, *Arcobacteraceae*, *Nitratiruptoraceae*, and *Nautiliaceae*. The values of ≥94.5% are shown in red. Intra-genus similarities are highlighted in grey.
(XLSX)

S6 Table Comparison of physiological characteristics of the genus *Helicobacter* based on the clade classification. (XLSX)

S1 File (TXT)

S2 File (PDF)

S3 File (PDF)
