# Supplementary figures and images for: Nitrosophilus alvini gen. nov., sp. nov., a hydrogen-oxidizing chemolithoautotroph isolated from a deep-sea hydrothermal vent in the East Pacific Rise, inferred by a genome-based taxonomy of the phylum “Campylobacterota”
Source: PLoS One. 2020 Dec 10;15(12):e0241366. doi: 10.1371/journal.pone.0241366 (PMC7728183; doi:10.1371/journal.pone.0241366)

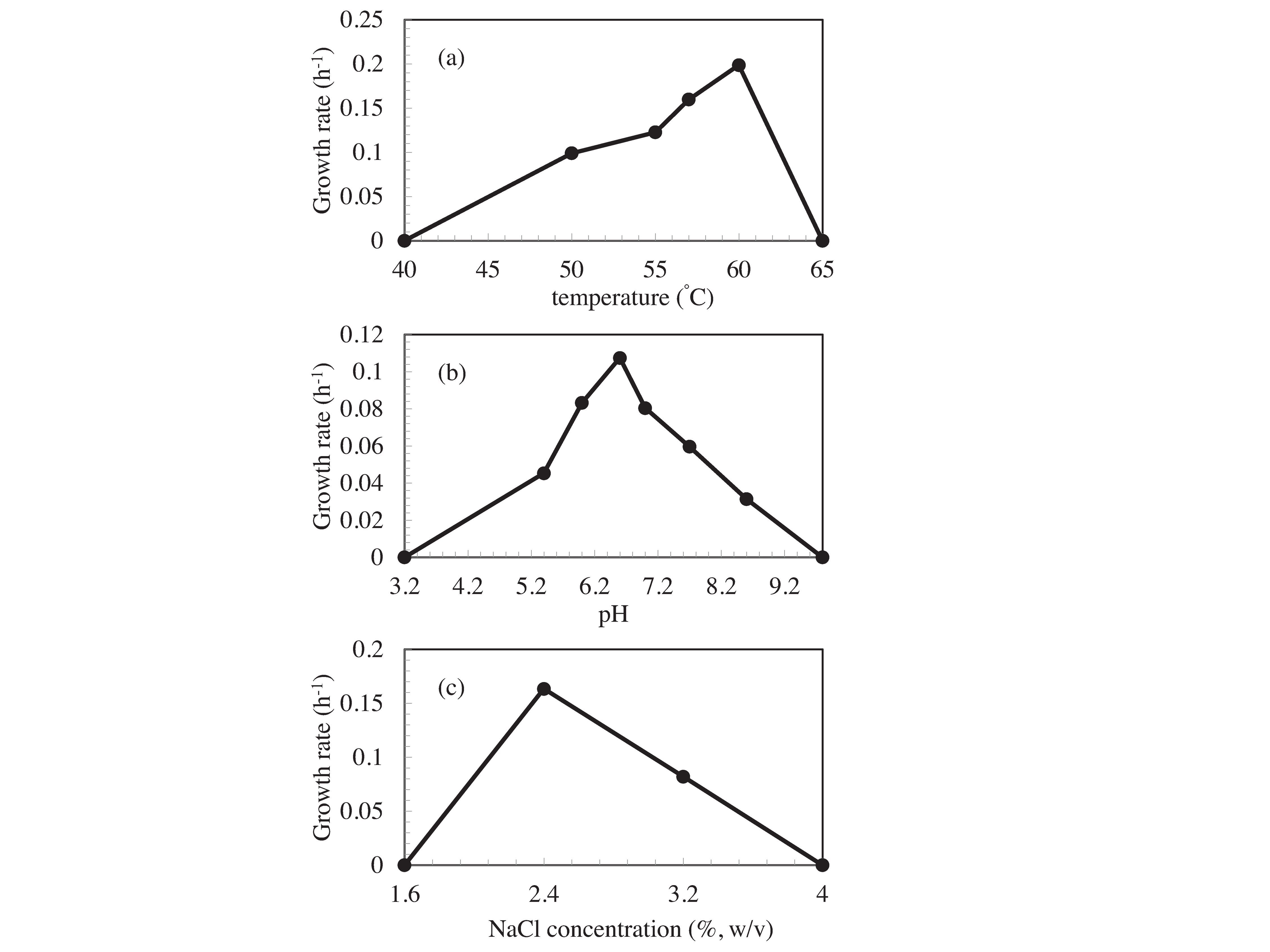

Supplement: S1 Fig — Growth rates of temperature (a), pH (b) and NaCl concentration (c) in MMJHS medium. (TIF) [file pone.0241366.s001.tif]

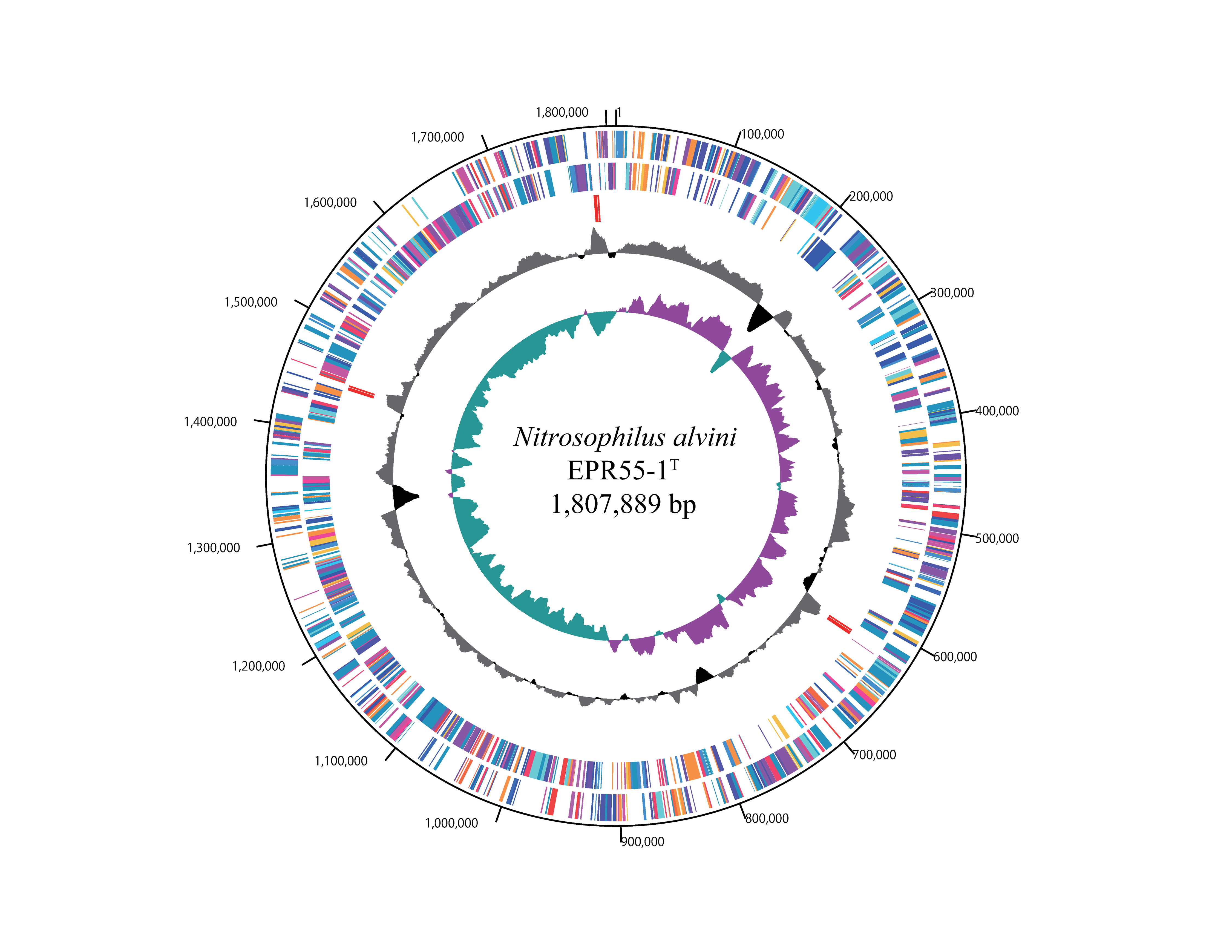

Supplement: S2 Fig — Tracks from inside to outside are as follows: GC skew, G + C content, rRNA, reverse strand CDS, and forward strand CDS (color by COG categories). (TIF) [file pone.0241366.s002.tif]

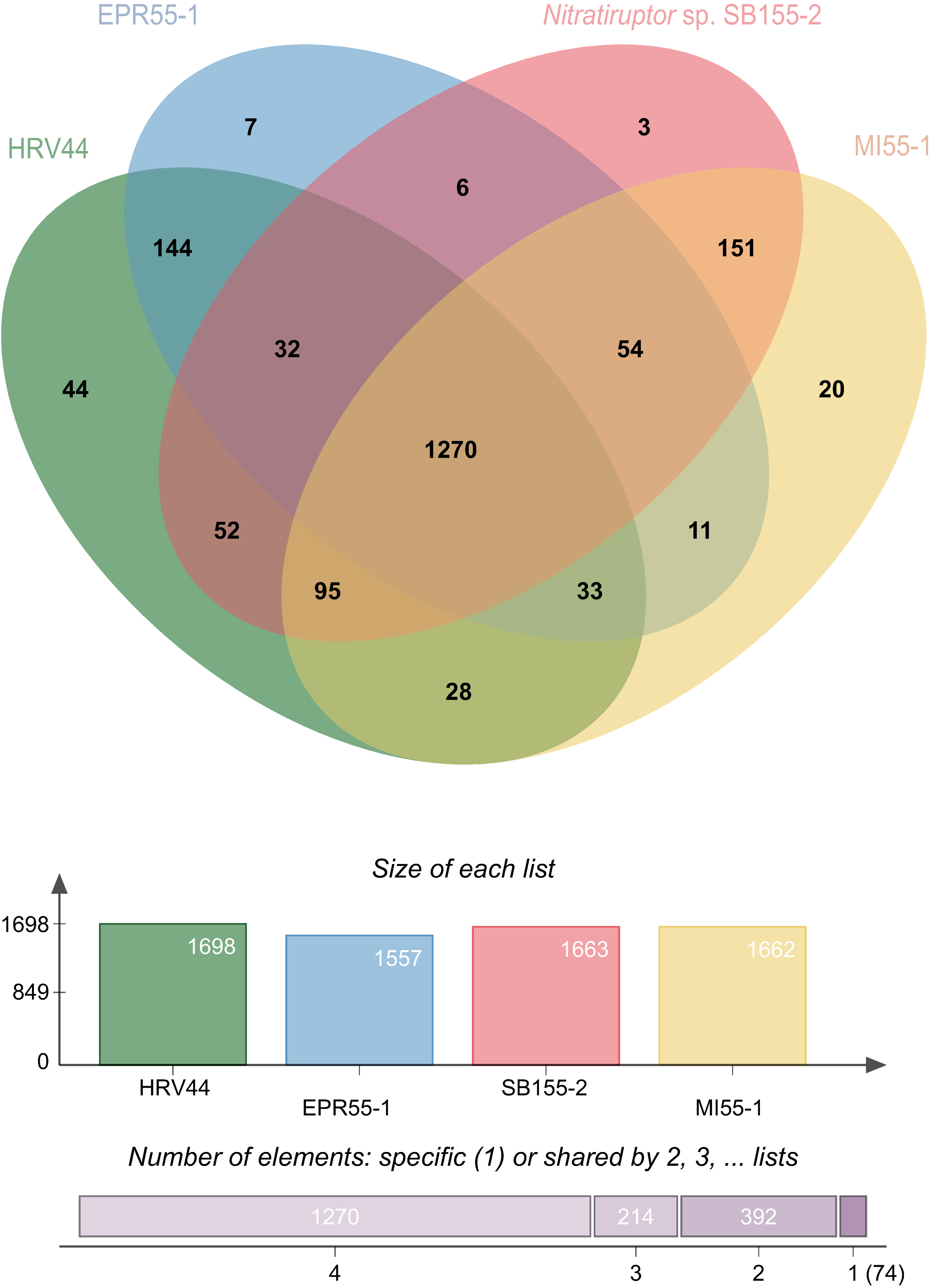

Supplement: S3 Fig — This Venn diagram represents shared or unique orthologous gene clusters between EPR55-1T, HRV44T, Nitratiruptor tergarcus MI55-1T and Nitratiruptor sp. SB155-2. (TIF) [file pone.0241366.s003.tif]

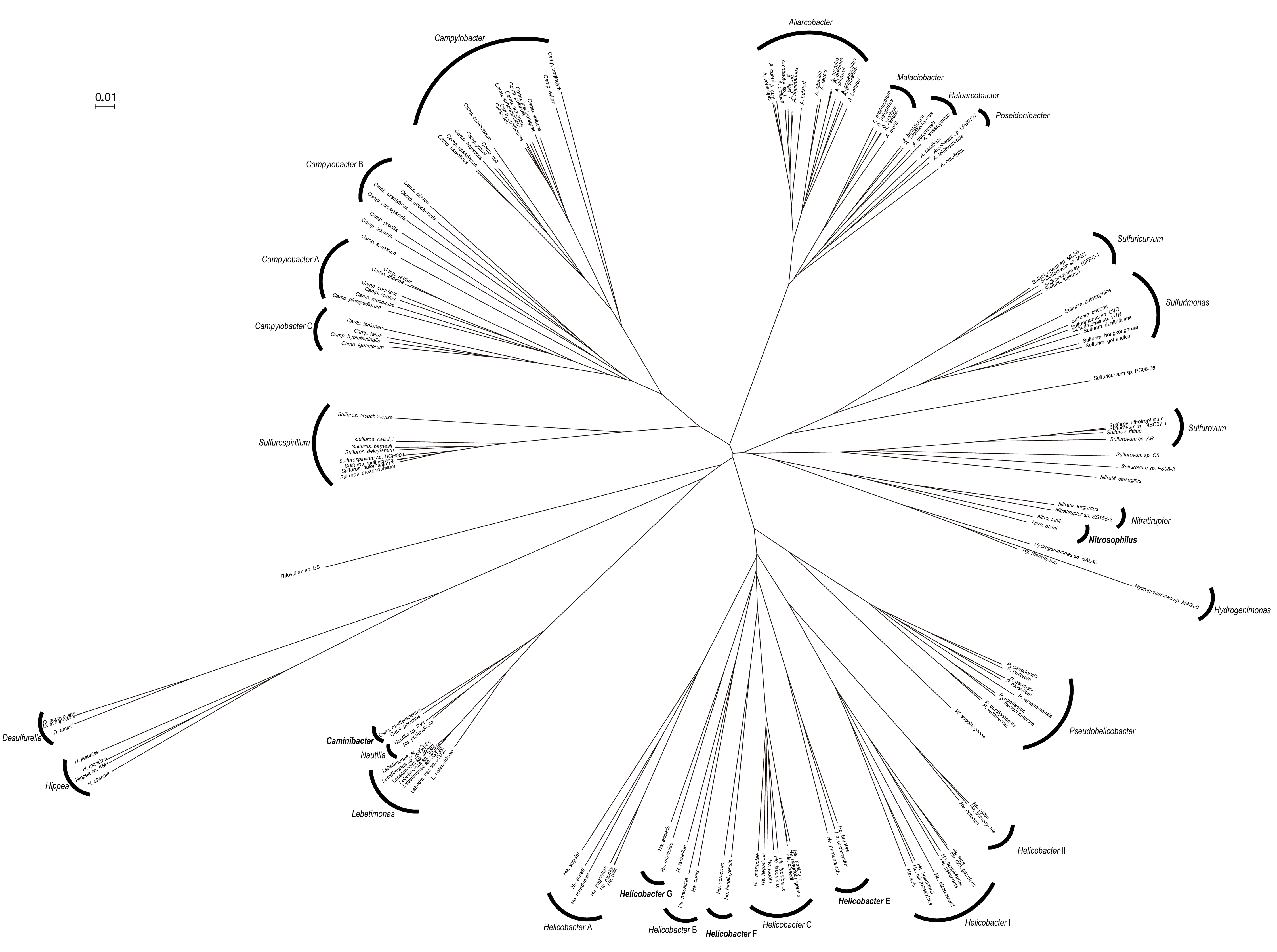

Supplement: S4 Fig — The phylogenomic tree was constructed based on 139 SCG protein sequences retrieved from 160 genomes belonging to “Campylobacterota”. The emended and newly proposed taxa in the phylum “Campylobacterota” (Nitrosophilus, Caminibacter, Helicobacter I, II, E, F, G, and Arcobacter) were shown in bold. (TIF) [file pone.0241366.s004.tif]

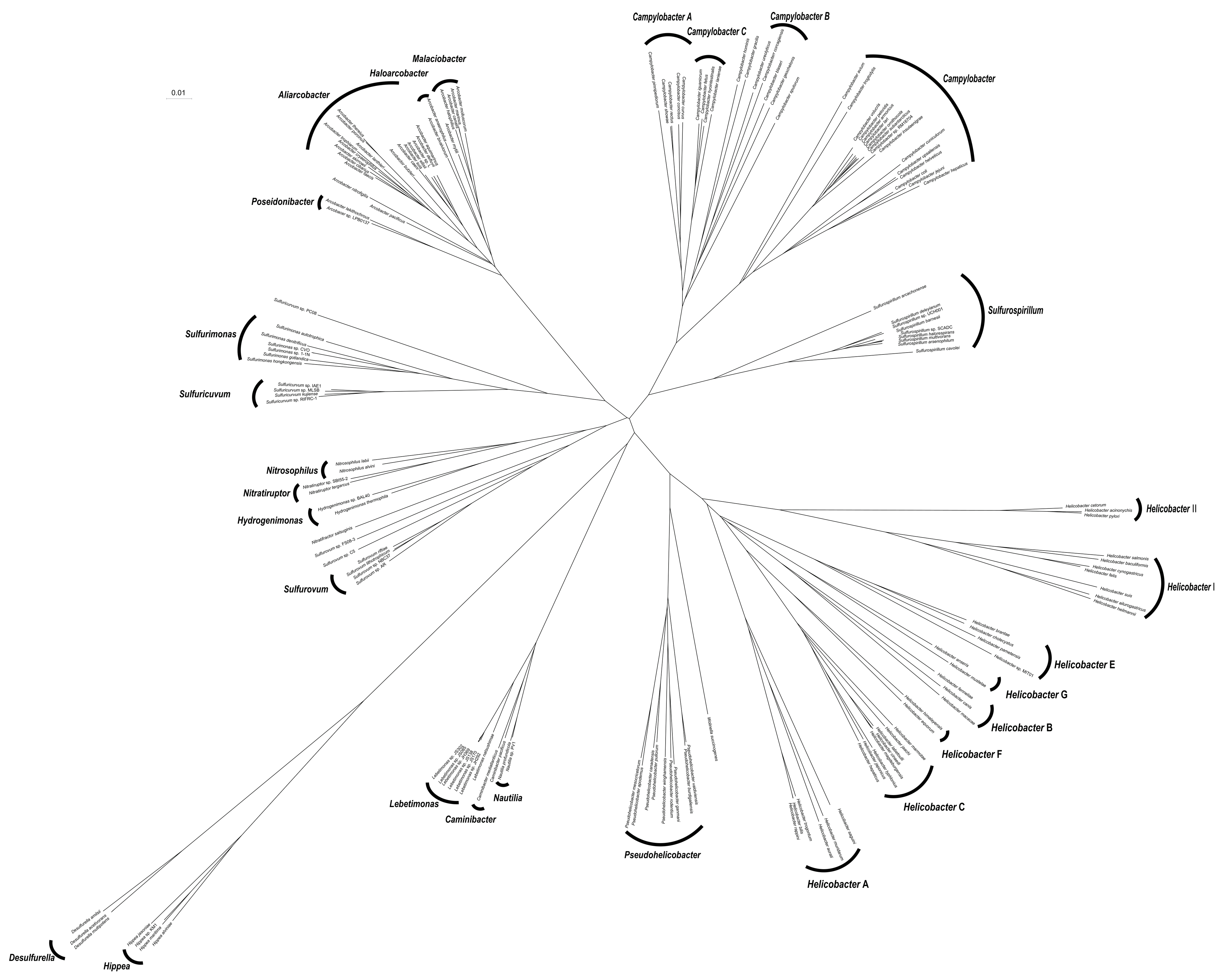

Supplement: S5 Fig — The phylogenetic tree was constructed based on amino acid sequences of MLSA genes (i.e. atpA, dnaK, glyA, gyrB, metG, pheS and tkt) retrieved from 154 members of “Campylobacterota”. Arcobacter cloacae F26, Arcobacter ebronensis CECT 8441, Arcobacter mediterraneus F156-34, Campylobacter mucosalis DSM 21682, Helicobacter bizzozeronii CIII-1, Hydrogenmonas sp. MAG80, and Thiovulum sp. ES were excluded because of lack of at least one MLSA gene sequence. (TIF) [file pone.0241366.s005.tif]
