# Supplementary material for: Nitrosophilus alvini gen. nov., sp. nov., a hydrogen-oxidizing chemolithoautotroph isolated from a deep-sea hydrothermal vent in the East Pacific Rise, inferred by a genome-based taxonomy of the phylum “Campylobacterota”
Source: PLoS One. 2020 Dec 10;15(12):e0241366. doi: 10.1371/journal.pone.0241366 (PMC7728183; doi:10.1371/journal.pone.0241366)
Supplement: S2 File — (PDF) [file pone.0241366.s013.pdf]

Korean Collection for Type Cultures (KCTC)
Korea Research Institute of Bioscience and Biotechnology (KRIBB)
181 Ipsin-gil, Jeongeup-si, Jeonbuk 56212, South Korea
Tel: +82-63-570-5602, FAX: +82-63-570-5609, E-mail: deposit@kribb.re.kr

Certificate of Deposit

Ref.: 20200273 | Date of issue: MAR 5, 2020

Taxonomic designation	:	***Nitratiruptor* sp.**
Accession number	:	**KCTC 15925**
Depositor(s)	:	Tomoo Sawabe
Strain code by the depositor(s)	:	**EPR55-1**

The above microorganism has been successfully deposited into the general collection of microorganism of the Korean Collection for Type Cultures (KCTC) and confirmed the identity of the microorganism under this KCTC number.
This microorganism will be available without restrictions for research and academic purposes in the publicly accessible section of the KCTC. It will be included in published and online catalogues after publication of this number by the authors.

Curator of Bacteria
Jung-Sook Lee Ph.D.

Telephone: +82-63-570-5618
Fax: +82-63-570-5609
E-mail: jslee@kribb.re.kr
