# Supplementary material for: Nitrosophilus alvini gen. nov., sp. nov., a hydrogen-oxidizing chemolithoautotroph isolated from a deep-sea hydrothermal vent in the East Pacific Rise, inferred by a genome-based taxonomy of the phylum “Campylobacterota”
Source: PLoS One. 2020 Dec 10;15(12):e0241366. doi: 10.1371/journal.pone.0241366 (PMC7728183; doi:10.1371/journal.pone.0241366)
Supplement: S3 File — (PDF) [file pone.0241366.s014.pdf]

JAPAN COLLECTION OF MICROORGANISMS

Microbe Division, RIKEN BioResource Research Center
3-1-1 Koyadai, Tsukuba, Ibaraki, 305-0074, Japan

Phone : +81 29 836 9556
Fax : +81 29 836 9561
E-mail : inquiry.jcm@riken.jp

平成31年4月18日

〒041-8611
北海道函館市港町3-1-1
国立大学法人 北海道大学
大学院水産科学研究院 海洋生物工学分野
美野 さやか 殿

国立研究開発法人 理化学研究所
バイオリソース研究センター
微生物材料開発室（JCM）

担当 伊藤 隆

室長 大熊 盛也

## JCM受託番号のお知らせ

この度は、当室の系統保存事業にご協力いただき、厚くお礼申し上げます。
先日ご寄託いただきました以下の微生物株について、当室では下記の番号で受託することになりました。本件に関するご照会にはこの番号をお書き添えくださるようお願い致します。
今後も本菌株を用いた研究を発表されましたら、情報をお寄せくださいますようお願い致します。

記

**JCM 32893** *Nitratiruptor pacificus*
<- S. Mino; Fac. of Fish. Sci., Hokkaido Univ., Japan; EPR55-1

以上
